# Eye diseases: the neglected health condition among urban slum population of Dhaka, Bangladesh

**DOI:** 10.1186/s12886-019-1043-z

**Published:** 2019-01-31

**Authors:** Ipsita Sutradhar, Priyanka Gayen, Mehedi Hasan, Rajat Das Gupta, Tapash Roy, Malabika Sarker

**Affiliations:** 1 BRAC James P Grant School of Public Health, BRAC University, Dhaka, Bangladesh; 2 Sajida Foundation, Dhaka, Bangladesh; 3 Institute of Global Health, University of Heidelberg, Heidelberg, Germany

**Keywords:** Refractive error, Cataract, Visual impairment, and Bangladesh

## Abstract

**Introduction:**

Globally, eye diseases are considered as one of the major contributors of nonfatal disabling conditions. In Bangladesh, 1.5% of adults are blind and 21.6% have low vision. Therefore, this paper aimed to identify the community-based prevalence and associated risk factors of eye diseases among slum dwellers of Dhaka city.

**Methods:**

The study was carried out in two phases. In the first phase, a survey was conducted using multistage cluster sampling among 1320 households of three purposively selected slums in Dhaka city. From each household, one family member (≥ 18 years old) was randomly interviewed by trained data collectors using a structured questionnaire. After that, each of the participants was requested to take part in the second phase of the study. Following the request, 432 participants out of 1320 participants came into the tertiary care hospitals where they were clinically assessed by ophthalmologist for presence of eye diseases. A number of descriptive and inferential statistics were performed using Stata 13.

**Result:**

The majority of total 432 study participants were female (68.6%), married (82.6%) and Muslim (98.8%). Among them almost all (92.8%) were clinically diagnosed with eye disease. The most prevalent eye diseases were refractive error (63.2%), conjunctivitis (17.1%), visual impairment (16.4%) and cataract (7.2%). Refractive error was found significantly associated with older age, female gender and income generating work. Cataract was found negatively associated with the level of education, however, opposite relationship was found between cataract and visual impairment.

**Conclusion:**

Our study provides epidemiologic data on the prevalence of eye diseases among adult population in low-income urban community of Dhaka city. The high prevalence of refractive error, allergic conjunctivitis, visual impairment, and cataract among this group of people suggests the importance of increasing access to eye care services.

**Electronic supplementary material:**

The online version of this article (10.1186/s12886-019-1043-z) contains supplementary material, which is available to authorized users.

## Background

Currently, eye diseases are considered as one of the major contributors of nonfatal disabling conditions in both high and low income countries [1]. The global burden of eye diseases was estimated to be 61.4 million DALYs, accounted for 4.0% of total DALYs [2]. Major contributors to the global burden of eye diseases are refractive errors (27.7 million DALYs), cataract (17.7 million DALYs), macular degeneration (9.3 million DALYs), glaucoma (4.7 million DALYs), trachoma (1.3 million DALYs), and vitamin A deficiency (0.6 million DALY) [2]. In addition, WHO estimated that globally 285 million people are visually impaired, of whom 39 million are blind [3]. The two main causes of visual impairment in the world are uncorrected refractive errors (42.0%) and cataract (33.0%) [4]. It is estimated that the South Asian region comprises a third of the world’s 45 million blind and the highest number of DALYs caused by eye diseases [5]. According to the National Blindness and Low Vision Survey of Bangladesh, 1.53% of adults, whose age is at least 30 years, are blind and 21.6% have low vision (presenting visual acuity of less than 6/12 in either one or both eyes) [6]**.** Cataract (73.4%) and refractive errors (18.9%) were found to be the main causes of visual impairment in Bangladesh [7]. Data from several studies suggest that insufficient food, substandard housing, and limited access to health care, education, water, and sanitation makes poor people more vulnerable to different diseases [8, 9]. Bangladesh Urban Health Survey (2013) reported that slum dwellers suffer from a poorer mental and physical health status than the rest of the population [10]. Therefore, it is imperative to ensure comprehensive eye care services at low or no cost for this vulnerable group of people.

In Bangladesh, few researches have been carried out on visual impairment and blindness, however, there is a paucity of investigations focusing on the prevalence and risk factors of common eye diseases among low-income population of Bangladesh. This paper unveiled the community based prevalence and associated risk factors of eye diseases among slum dwellers of Dhaka city. The findings of this study will provide strong insight to the policy makers and public health professionals about magnitude of different eye diseases, which, in turn will help them to design community-based programs to address the eye care needs of vulnerable slum population.

## Methods

This study was carried out in two phases. In the first phase, a survey was conducted using multistage cluster sampling among 1320 households of three purposively selected slums in Dhaka city (Shabujbag slum, Mirpur slum and Mohammadpur slum). These slums were selected due to the presence of well-known tertiary eye care facility within the close proximity. The population size of the slums was taken into account while selecting 520 households from Shabujbag slum and 400 households from Mirpur and Mohammadpur slum each. Then from each household, one family member of at least 18 years age was randomly selected for the interview. Trained data collectors were assigned to collect socio-demographic and eye related information of the respondents using a structured questionnaire (Additional file 1). After that, each of the participants was requested to take part in the second phase of the study and their participation was voluntary. All of the 1320 household participants were given referral cards for free eye check up at any one of the three-selected tertiary eye care facilities (Ad-Din Women’s Medical College and Hospital, BNSB Dhaka Eye Hospital, VARD Eye Hospital). Finally, 432 out of the 1320 participants (33%) came into the tertiary care hospital for free eye examination. The participants were clinically examined by trained opthalmologists. The opthalmologists filled up a form that narrates the results of the clinical examination (Additional file 2).

Before the clinical examination trained data collectors conducted interview with the respondents using a structured questionnaire to cross check the socio-demographic information that was collected before.

### Clinical examination

In this study eye diseases were diagnosed clinically by qualified medical professionals (ophthalmologists). All 432 participants who attended the tertiary eye care facilities were examined for the presence of eye diseases after obtaining informed written consent. The eye examination was systematically performed using guideline adopted from “The American Academy of Ophthalmology”, which included refraction test, examination of visual acuity, pupils, extra ocular motility and alignment, intraocular pressure, external examination, slit-lamp examination and fundoscopy [11].

For all eye diseases, we presented data for either eye. Refractive error was assessed using autorefractor and 6/6 vision was considered as emetropia. Participants were considered as having myopia or hypermetropia when spherical equivalent refractive error was less than − 0.50 diopters or more than + 0.50 diopters respectively. Data on visual acuity was reported as the presenting visual acuity obtained through presenting correction. The definitions for visual impairment, low vision and blindness used in this study follow those given in the international statistical classification of diseases, injuries and causes of death, 10th revision (ICD-10) [12] (Table 1).

**Table 1:** Categories of severity of visual impairment according to the International Statistical Classification of Diseases (ICD-10)

Category	Presenting distance visual acuity	
	Worse than:	Equal to or better than:
0 Mild or no visual impairment	N/A	6/18
1 Moderate visual impairment	6/18	6/60
2 Severe visual impairment	6/60	3/60 or 6/120
3, 4, 5 Blindness	3/60 or 6/120	1/60

### Statistical analysis

Data was entered using CSPro version 6.0. After cleaning, data was transferred into Stata software (version 13.0 for Windows, 4905 Lakeway Drive, College Station, Texas 77,845 USA) for statistical analysis. Descriptive analysis was performed to estimate the prevalence of different eye diseases and was presented as frequency and percentage. Cross tabulation was performed to see the distribution of eye diseases among study participants across different age, gender, family income, educational level, occupation, and wealth index. Then these variables were tested for associations with different eye diseases in bivariate analysis using the Fisher’s exact test or Chi-square test as appropriate. Variables associated with different eye diseases in bivariate analysis were further tested in stepwise multivariable logistic regression model. A two-tailed *P*-value less than 0.05 was considered statistically significant.

### Findings

#### Study population

A total of 432 respondents from three urban slums of Dhaka city participated in the study. The results, as shown in Table 2, indicate that the mean age of the study participants was 37.9 years (SD ± 13.30) and just over half of them were from 18 to 40 years of age (54.2%). Majority of the study participants were female (68.6%), married (82.6%) and almost all of them were Muslim (98.8%). On average, family income of our study participants was 13,270 BDT (SD: ± 8715) per month. Nearly half of them received no formal education (43.5%) and almost similar number of respondents were involved in income generating work (37.5%).

**Table 2:** Socio-demographic information of 432 adult (≥ 18 years) slum dwellers of Dhaka, Bangladesh

Variables	*n* (%)	Variables	*n* (%)
Age (Mean: 37.96 years, SD: ± 13.30)		Educational status	
18–40 years	234 (54.2)	No formal education	188 (43.5)
> 40 years	198 (45.8)	Some formal education	244 (56.5)
Gender		-Primary education	155 (35.88)
Male	135 (31.3)	-Secondary education	55 (12.73)
Female	297 (68.8)	-SSC/HSC/Equivalent	24 (5.56)
Marital status		-Graduation and above	10 (2.31)
Married	357 (82.6)	Occupational status	
Unmarried	28 (6.5)	Income generating	162 (37.5)
Widowed/Separated/Divorced	47 (10.9)	-Wage worker	88 (20.4)
Religion		-Self employed	29 (6.7)
Muslim	427 (98.8)	-Garment worker	23 (5.3)
Hindu	5 (1.2)	-Service	22 (5.1)
Family income (Mean: 13270, SD: ± 8715) BDT per month		Non-income generating	270 (62.5)
≤ 12,000 BDT per month	238 (55.1)	-Homemaker	181 (41.9)
> 12,000 BDT per month	194 (44.9)	-Other (Student, retired, unemployed etc.)	89 (20.6)

#### Prevalence of eye disease

Out of the 432 participants, 401 (92.8%) were diagnosed with any form of eye diseases. Among them, 237 (59.1%) participants had only one eye disease, 146 (36.4%) had two different eye diseases and 18 (4.5%) of the participants were diagnosed with three different types of eye illnesses. Refractive error, allergic conjunctivitis, visual impairment, and cataract were found as the most prevalent eye diseases in our study (Table 3).

**Table 3:** Prevalence of eye diseases among 432 adult (> 18 years) slum dwellers of Dhaka, Bangladesh*

Inflammatory		Non-inflammatory			
Name of eye disease	Prevalence *n* (%)	Name of eye disease	Prevalence *n* (%)	Name of eye disease	Prevalence *n* (%)
Conjunctivitis	74 (17.1)	Refractive error	273 (63.2)	Posterior capsule opacification	2 (0.2)
− Allergic	− 65 (15.0)				
− Bacterial & Chronic	− 9 (2.1)				
Dacryocystitis	14 (3.2)	Visual impairment		Congenital iris coloboma	2 (0.5)
		− Either eye	− 71 (16.4)		
		− Right eye	− 56 (13.0)		
		− Left eye	− 53 (12.3)		
		Cataract	31 (7.2)	Glaucoma	2 (0.5)
Blepharitis	6 (1.4)	Dry eye syndrome	14 (3.2)	Bilateral/Alternate divergent strabismus	2 (0.5)
Others (Scleritis, uveitis, keratitis)	3 (0.7)	Pterygium	13 (3.0)	Muscae volitantes	1 (0.2)
		Corneal ulcer/scar	6 (1.4)	Congenital microcornea	1 (0.2)
		Pseudophakia	4 (1.0)	Others (foreign body, trauma, phthisis bulbi)	27 (6.3)

*Cumulative percentage is > 100% due to presence of multiple eye disease in one person

Refractive error had the highest prevalence among all eye illnesses with a frequency of 273 (63.2%) (Table 3). Of these 273 patients, the most prevalent refractive error was presbyopia 144 (33.3%), followed by hypermetropia 15 (3.5%) (Table 4). Conjunctivitis was found as the second most prevalent eye diseases among our study population with a frequency of 74 (17.1%). Among our respondents, 31 (7.2%) were diagnosed as having cataract. Moreover, a total of 71 (16.4%) subjects were classified as having visual impairment (presenting visual acuity< 6/18, including both low vision and blindness) in either eye of which 56 (13.0%) and 53 (12.3%) respondents were visually impaired in right and left eye respectively.

**Table 4:** Prevalence of different types of refractive error among 432 adult (≥ 18 years) slum dwellers of Dhaka, Bangladesh^a^

Type of refractive error	Prevalence *n* (%)
Presbyopia	144 (33.3)
Hypermetropia	15 (3.5)
Myopia	7 (1.6)
Anisometropia	7 (1.6)
Astigmatism	12 (2.8)
Non-specific refractive error	145 (33.6)

^a^The cumulative percentage is not 100%, as there are individuals diagnosed with more than one type of refractive error

#### Risk factors

Table 5 presents the summary statistics for associations of different eye diseases (refractive error, visual impairment, and cataract) with socio-demographic variables (age, gender, education, occupation and family income). The prevalence of refractive error and cataract increased significantly with age at 5% level of significance. Subjects having more than 40 years of age were 3.02 times more likely to develop refractive error (*p < 0.001*) and 26.42 times more likely to develop cataract (*p = 0.002*). The prevalence of refractive error was also found to be influenced by gender and occupation of the study participants. The result of our study suggested that, at 5% level of significance, women were 2.92 times more likely to develop refractive error (*p < 0.001*) compared to men. People who were involved in income generating work also had significantly higher prevalence of refractive error (Adjusted OR = 1.70, 95% CI: 1.00–2.88, *p = 0.048*). The prevalence of cataract was observed to be associated with the study participants’ level of education. People having cataract were less likely to receive any formal education (Adjusted OR = 3.37, 95% CI: 1.28–8.89, *p* = 0.014). Nevertheless, cataract was found to have positive association with visual impairment (Adjusted OR = 7.33, 95% CI: 2.78–19.29, *p < 0.001*).

**Table 5:** Multivariable logistic regression models assessing the adjusted odds ratio (95% CI) of eye diseases

		Refractive error		Visual impairment		Cataract	
Variables		Adjusted OR (95% CI)	*P value*	Adjusted OR (95% CI)	*P value*	Adjusted OR (95% CI)	*P value*
Age (y)	> 40 years						
	≤ 40 years	3.02 (1.90–4.78)	< 0.001*	1.81 (0.85–3.88)	*0.122*	26.42 (3.49–200.06)	0.002*
Sex	Male						
	Female	2.92 (1.65–5.16)	< 0.001*	0.75 (0.30–1.86)	*0.539*	0.37 (0.12–1.14)	*0.085*
Education	Some formal education						
	No formal education	0.79 (0.50–1.25)	*0.324*	1.48 (0.74–2.96)	*0.255*	3.37 (1.28–8.89)	0.014*
Occupation	Non-income generating						
	Income generating	1.70 (1.00–2.88)	0.048*	0.52 (0.22–1.21)	*0.131*	0.60 (0.20–1.78)	*0.365*
Family income (BDT)	≥ 12,000 per month						
	< 12,000 per month	0.99 (0.65–1.49)	*0.976*	0.65 (0.35–1.23)	*0.195*	1.01 (0.45–2.24)	*0.975*
Refractive error	Yes			0.70 (0.34–1.43)	*0.332*		
	No			7.33 (2.78–19.29)	< 0.001*		
Cataract	Yes						
	No			0.75 (0.30–1.86)	*0.539*		

*P value < 0.05 is statistically significant

## Discussion

To the best of our knowledge, this was the first reported study in Bangladesh providing data on the presence of clinically diagnosed eye diseases in low-income urban population of Dhaka, Bangladesh. The major findings of this study was detection of high prevalence of different eye diseases which would have been undiagnosed in absence of this active screening method.

Refractive error was found as the most prevalent eye disease among our study population (63.2%). This prevalence is higher than that found in the National Blindness and Low Vision Survey of Bangladesh (42.7%) (1999–2000) [7]. It is possible that prevalence of refractive error has increased in Bangladesh during this 14-years period of time as a result of possible cohort effect documented in other studies [13]. However, as there are ample inconsistencies in the methods used in these two studies, further investigation is necessary to decide whether the prevalence of refractive error is higher in low-income urban population than that in nationally representative sample and if so, to find out the potential reasons for such a high prevalence. Conjunctivitis was found as the second most prevalent eye diseases among our study population and most common type of conjunctivitis identified was allergic conjunctivitis (15.0%). Allergic conjunctivitis is one of the most common inflammatory disorders of anterior chamber of the eye, which has been considered the epidemics of the twenty-first century [14]. Comparison with the similar aged population was not possible due to the scarcity of international data, estimating the prevalence of ocular allergies within adult populations. However, our finding is similar to the prevalence of allergic rhino conjunctivitis (2.2–24.2% in children and 4.5–45.1% in adolescents) estimated by the International Study of Asthma and Allergies in Childhood (ISAAC). Phase III of this study involved 1, 93,404 schoolchildren aged 6–7 years from 37 countries and 3, 04,679 adolescents aged 13–14 years from 56 countries [15]. Unlike to refractive error, the prevalence of allergic conjunctivitis was considerably lower among our study population (15.0%) than that in other Asian countries (India, Japan, Thailand, and Singapore) where up to 30% of adults generally suffer from this disease [16].

The overall prevalence of cataract in our study participants was 7.2%. However, this prevalence was 15.2% among the respondents above 40 years of age. The prevalence of visual impairment in our study population (12.96%) was slightly higher than the estimated prevalence of visual impairment (VA < 6/18) in WHO South-East Asian Region (Bangladesh, Democratic Republic of Timor-Leste, Indonesia, Myanmar, Nepal, India excluded) which is 9.8% [17]. This difference could be due to the social, economic and political exclusion experienced by our study population. According to Baker, in 2007, only 7.3% of the slum dwellers in Dhaka city had access to public health care facilities [18]. This also might be due to the fact that, the programs developed and carried out by different organizations (NGOs, donors, government) are frequently unable to reach this underprivileged group of people living in the urban slums [19].

It is well evident that eye diseases impose a detrimental effect on the quality of life of affected individuals [20]. Uncorrected refractive error significantly reduces the self-reported visual ability as well as functional ability like mobility, driving, performing near vision tasks of an individual which ultimately affect his/her quality of life [21]. Patients with allergic conjunctivitis can experience significant impairment in their quality of life since moderate angina and the condition worsens during an acute episode for approximately 46% of the patients [22]. Visual impairment and age-related cataract have potential to act as independent risk factors for increased mortality in older persons [23]. Visual impairment alone also significantly reduce a person’s physical and mental well-being [24], though, the mental impact of visual impairment is much greater than its physical impact [25]. Individuals having visual impairment face difficulty to maintain mobility, suffer from depression and experience compromised self-ranking health status that is similar to a medical condition like stroke [23].

In this study, although we offered free checkup of eye diseases, the low number of participant turnout in the health facilities and a high number of untreated eye conditions indicate that eye health is not a priority in the low-income urban community in Bangladesh. Individuals living in urban slums are already burdened with a miserable set of circumstances along with lack of adequate knowledge about the origin of disease and appropriate measures for cure [26]. The situations further worsen as a result of their restricted access to societal assets like educational institutes, healthcare facilities, safe water source and basic sanitation [27]. Studies suggest an urgent need for health education and access to eye care services for these underprivileged slum populations to ameliorate this situation [28]. A study found that visually impaired persons who received support from friends and family members could adapt to vision loss more effectively, had less depressive symptoms, and higher life satisfaction [29]. Therefore, social support is also vital to improve the eye health status of this slum population.

As reported in other populations, increased age was positively associated with different eye diseases in our study like refractive error and cataract. It is evident that refractive index of the human eye lens is inhomogeneous [30] though the actual underlying principle for this opinion is yet a matter of debate. Refractive index distribution changes significantly with age, where main change is characterized by a decrease in the nuclear refractive index [31]. With increasing age, many types of modifications occur in the crystalline proteins of the human lens like protein denaturation and oxidation, which are responsible for age-related hardening and opacification of the lens nucleus and development of age-related cataract [32].

Our study shows a significantly higher prevalence of refractive error in women as compared to men which is similar to other studies [33]. The association of gender and refractive error has not been well established. This may be because women’s eyes have different axial length and anterior chamber depth than those of men and hence a higher probability of being affected by refractive error [34]. Environmental factor like close work and genetic factor also might be the reason for the divergence in axial length between men and women [35].

In our study cataract was significantly associated with the educational level of study participants. This finding is in accordance with previous studies like Framingham Eye Study [36] and lens opacities case-control study [37]. As well, in an age-adjusted analysis of a survey data carried out in India (Punjab), the prevalence of cataract was significantly higher in the group having no formal education [38]. Low education might have no apparent biological relation with cataract development. Confounding factors such as nutritional deficiency (Protein, vitamin A, niacin, thiamin and riboflavin deficiency) [39], UV-B exposure [40] and lifestyle related factors like smoking [41] might explain this association, therefore, further investigation needs to be carried out. Like previous research findings, cataract was found to be significantly associated with visual impairment in our study after adjusting for other potential confounders. This finding is also supported by data from prior studies that identified cataract as the most important causes of visual impairment globally [4] and in Bangladesh [6].

Previous studies show that awareness and knowledge of common eye diseases in developing countries are significantly less among the group of people having lack of formal education, low socioeconomic status and among female [42]. This means that increasing opportunity for better earning, basic education and raising awareness of eye diseases could lead to an improvement in understanding and acceptance of the importance of early detection and treatment of eye diseases. In this way, it is possible to lessen the cost of eye care, reduce the incidence of visual impairment and thus improve the quality of life.

The strength of this study was its standardized protocol and diagnosis of eye diseases by qualified ophthalmologists. However, the major limitation of our study was the low participation of the study population from household survey to tertiary eye care facilities. Another limitation of this study is potential over estimation of the clinically diagnosed eye diease prevalence due to the selection bias.

## Conclusion

Our study provides epidemiologic data on the prevalence of clinically diagnosed eye diseases among the adult population in low-income urban community of Dhaka city. The high prevalence of refractive error, allergic conjunctivitis, cataract, and visual impairment suggests the importance of provision for eye care services to this group of people. In Bangladesh, ophthalmologists provide treatment for different eye illnesses at hospital settings, while BRAC and few other organizations provide eye care at community settings. Basic eye care including screening of the refractive errors can be implemented at the primary level by the community health workers at an affordable cost. There should also be community awareness program to promote the information on importance of early eye care to prevent long term consequences. Large population based longitudinal study is also needed to identify other behavioural, environmental and genetic risk factors that might be unique for Bangladeshi population.

## Additional files

Additional file 1: Survey Questionnaire. This questionnaire was used to collect data on participants’ socio-demographic information, household related information, and eye care related information. (DOCX 128 kb)

Additional file 2: Clinical examination form This form was used to collect data on participants’ eye examination result and clinical diagnosis of eye disease. (DOCX 1713 kb)

## Abbreviations

BDT: Bangladeshi Taka
BNSB: Bangladesh National Society for the Blind
DALY: Disability-Adjusted Life Year
UV-B: Ultra violet-B
VARD: Voluntary Association for Rural Development
WHO: World Health Organization
