# Supplementary material for: Eye diseases: the neglected health condition among urban slum population of Dhaka, Bangladesh
Source: BMC Ophthalmol. 2019 Jan 31;19:38. doi: 10.1186/s12886-019-1043-z (PMC6357461; doi:10.1186/s12886-019-1043-z)
Supplement: Supplementary file 1 — Survey Questionnaire. This questionnaire was used to collect data on participants’ socio-demographic information, household related information, and eye care related information. (DOCX 128 kb) [file 12886_2019_1043_MOESM1_ESM.docx]

SURVEY Questionnaire

participant informed Consent FORM

Household interview

**Accessibility of Eye Care Service in an Urban Setting in Bangladesh**

Dear Participant,

Greetings, I am _________________________ and this is *(If there are two interviewers)*. I am/we are working in a research project under BRAC James P Grant School of Public Health, BRAC University.

We are conducting a study on prevalence of eye problems in the community and heath seeking behavior for eye problems. In this study we hope to learn the prevalence of different eye problems existing in different age groups and where people seek eye care for these problems.

You are invited to participate in this study. You will have to answer some questions regarding eye problems and eye care. The interview will not take more than 15-20 minutes. Your responses will be kept confidential and will not be used for any other purpose other than research. You can ask for any clarification of any question and can withdraw from the study anytime you want. If you have any current eye problem of any kind, we will refer you to a specialized eye care facility, where you will receive eye examination. This eye examination will be conducted by qualified eye specialist. It will be non-invasive, risk-free and free of cost.

Do you have any further inquiry about this study? Yes No

(*If “Yes” please answer to any other inquiry of the participant)*

Do you agree to participate in this study? Yes No

**SECTION 01: HOUSEHOLD MEMBER INFORMATION**

| Q1_01 | Q1_02 | Q1_03 | Q1_04 | | Q1_05 | Q1_06 | Q1_07 |
| --- | --- | --- | --- | --- | --- | --- | --- |
| Mem No. | Name | Gender Code  Male = 1 Female= 2  Other= 3 | Age | | Marital Status** (Please Check Code Box) | Educational Qualification ***  (Please Check Code Box) | Occupation▼  (Please Check Code Box) |
|  |  |  | Year | Month |  |  |  |
| [__] |  | [__] | [__\|__]-[__\|__] | | [__\|__] | [__\|__] | [__\|__] |
| [__] |  | [__] | [__\|__]-[__\|__] | | [__\|__] | [__\|__] | [__\|__] |
| [__] |  | [__] | [__\|__]-[__\|__] | | [__\|__] | [__\|__] | [__\|__] |
| [__] |  | [__] | [__\|__]-[__\|__] | | [__\|__] | [__\|__] | [__\|__] |
| [__] |  | [__] | [__\|__]-[__\|__] | | [__\|__] | [__\|__] | [__\|__] |
| [__] |  | [__] | [__\|__]-[__\|__] | | [__\|__] | [__\|__] | [__\|__] |
| [__] |  | [__] | [__\|__]-[__\|__] | | [__\|__] | [__\|__] | [__\|__] |
| [__] |  | [__] | [__\|__]-[__\|__] | | [__\|__] | [__\|__] | [__\|__] |
| [__] |  | [__] | [__\|__]-[__\|__] | | [__\|__] | [__\|__] | [__\|__] |

| **Marital Status Code | ***Educational Qualification Code | ▼Occupation Code | |
| --- | --- | --- | --- |
| 01 = Married | 01 = Never attended school | 01= Day Labour | 09= Doctor (MBBS) |
| 02 = Unmarried | 02= Primary (class 1-5) | 02= Rickshaw / Van puller | 10=Doctor (Alternative medicine) |
| 03 = Widowed | 03= Secondary (class 6-10) | 03= Businessmen/ shop owner | 11=House owner |
| 04 = Divorced | 04= SSC/Dakhil/Equivalent | 04= Vendor | 12=Housewife |
| 05 = Separated | 05= HSC/Fazil/Equivalent | 05= Driver (Motor vehicle) | 13=Student |
| 06= Other Please specify | 06= Graduate | 06= Garments worker (Sewing) | 14=Retired |
|  | 07= Masters | 07= Garments worker (Administration) | 15= Unemployed |
|  | 08= Others (Please specify) | 08= Service holder (Govt./Private/ NGO) | 16= Others (please specify)  [­­­­­­­­­­­­­­­­­­­­_________________] |

| Random No.: [_0_\|_._\|__\|__\|__\|__\|__] | | | | | | | | | |
| --- | --- | --- | --- | --- | --- | --- | --- | --- | --- |
| Member ≥ 15 year | [__] | [__] | [__] | [__] | [__] | [__] | [__] | [__] | [__] |
| Tick the selected member |  |  |  |  |  |  |  |  |  |

**SECTION 02: HOUSEHOLD INFORMATION**

| **Code** | **Question** | | **Response codes** | **Answer** |
| --- | --- | --- | --- | --- |
| URBRUR | **The household in Rural or Urban area?** | | Urban = 1  Rural = 2 | [__] |
| W01 | **What is the main source of drinking water for members of your household?** | | | [__\|__] |
|  | Piped water  Piped into dwelling =11  Piped to yard / plot= 12  Public tap/standpipe =13  Tube well or borehole = 21 | Dug well  Protected well = 31  Unprotected well = 32  Water from spring  Protected spring = 41  Unprotected spring = 42 | | Rainwater = 51  Tanker truck = 61  Cart with small tank = 71  Surface water (river/dam/lake/pond/stream/canal/ irrigation channel) = 81  Bottled water = 91  Other = 96 |
| W02 | **What kind of toilet facility do members of your household usually use?** | | | [__\|__] |
|  | Flush or pour flush toilet  Flush to piped sewer system= 11  Flush to septic tank= 12  Flush to pit latrine= 13  Flush to somewhere else= 14  Flush, don't know where= 15 | Pit latrine  Ventilated improved pit latrine= 21  Pit latrine with slab = 22  Pit latrine w/out slab/open pit= 23 | | Composting toilet= 31  Bucket toilet= 41  Hanging toilet/hanging latrine = 51  No facility/bush/field= 61  Other = 96 |
| W03 | **Do you share this toilet facility with other households?** | | Yes = 1  No = 2 | [__] |

| **Code** | **Question** | **Response codes** | **Answer** |
| --- | --- | --- | --- |
| **Does your household have:** | | | |
| W04 | Electricity? | Yes = 1 No = 2 | [__] |
| W05 | A radio? | Yes = 1 No = 2 | [__] |
| W06 | A television? | Yes = 1 No = 2 | [__] |
| W07 | A mobile telephone? | Yes = 1 No = 2 | [__] |
| W08 | Anon-mobile telephone? | Yes = 1 No = 2 | [__] |
| W09 | A refrigerator? | Yes = 1 No = 2 | [__] |
| W10 | An almirah/wardrobe? | Yes = 1 No = 2 | [__] |
| W11 | A table? | Yes = 1 No = 2 | [__] |
| W12 | A chair? | Yes = 1 No = 2 | [__] |
| W13 | An electric fan? | Yes = 1 No = 2 | [__] |
| W14 | ADVD/VCD player? | Yes = 1 No = 2 | [__] |
| W15 | A water pump? | Yes = 1 No = 2 | [__] |

| **Code** | **Question** | | **Response codes** | | **Answer** |
| --- | --- | --- | --- | --- | --- |
| W16 | **What type of fuel dose your household mainly use for cooking?** | | | | [__\|__] |
|  | Electricity= 01  LPG = 02  Natural gas =03  Bio gas = 04 | Kerosene= 05  Coal, lignite= 06  Charcoal= 07  Wood= 08 | | Straw/shrubs/grass= 09  Agricultural crop= 10  Animal dung= 11  No food cookedin household= 95 | |
| W17 | **What is the main material of the floor of the household?** | | | | [__\|__] |
|  | Natural Floor:  Earth/Sand = 11  Rudimentary Floor:  Wood Planks = 21  Palm /Bamboo = 22 | Finished Floor:  Parquet or polished wood = 31  Ceramic = 33  Cement =34  Carpet = 35 | | Other= 96 | |

| **Code** | **Question** | | **Response codes** | | **Answer** |
| --- | --- | --- | --- | --- | --- |
| W18 | **What is the main material of the roof of the household?** | | | | [__\|__] |
|  | Natural Roofing:  No Roof = 11  Thatch/ Palm Leaf = 12  Rudimentary Roofing:  Palm /Bamboo = 22  Wood Planks = 23  Card Board = 24 | Finished Roofing:  Tin = 31  Wood = 32  Ceramic Tiles =34  Cement= 35  Roofing Shingles = 36 | | Other = 96 | |
| W19 | **What is the main material of the external walls in your household?** | | | | [__\|__] |
|  | Natural Walls:  No Walls = 11  Cane/ Palm / Trunks = 12  Dirt = 13  Rudimentary Walls:  Bamboo with Mud = 21  Stone with Mud = 22  Plywood= 24  Card Board = 25 | Finished Walls:  Tin = 31  Cement = 32  Stone with lime / Cement =33  Bricks= 34  Wood Planks / Shingles = 36 | | Other = 96 | |

| **Code** | **Question** | **Response codes** | **Answer** |
| --- | --- | --- | --- |
| W20 | **How many rooms in this household are used for sleeping?** | | Room No: [__\|__] |
| **Does any member of this household own:** | | | |
| W21 | **An auto bike?** | Yes = 1 No = 2 | [__] |
| W22 | **A bicycle?** | Yes = 1 No = 2 | [__] |
| W23 | **A rickshaw / van?** | Yes = 1 No = 2 | [__] |
| W24 | **A motor cycle or motor?** | Yes = 1 No = 2 | [__] |
| W25 | **How much land does your household own (Other than the homestead land)?** | 95 or more Acres = 95 | Acres: [__\|__] |
| W26 | **Does ant member of this household have a bank account?** | Yes = 1 No = 2 | [__] |
| W27 | **How many people live in your house?** | | People: [__\|__] |
| W28 | **Are there any domestic servants**  **(Non-family members) working in your household?** | Yes = 1 No = 2 | [__] |

| **Code** | **Question** | **Response codes** | **Answer** |
| --- | --- | --- | --- |
| W29 | **Does this household own any livestock, herds, other farm animals, or poultry?** | Yes = 1 No = 2 | [__] |
| **How many of the following animals does this household own?** | | | |
| W30 | **Buffaloes / Cows?** | None = 00  95 or more = 95 | [__\|__] |
| W31 | **Milk cows or bulls?** |  | [__\|__] |
| W32 | **Goats or sheep?** |  | [__\|__] |
| W33 | **Chickens or ducks?** |  | [__\|__] |

**Section 03: Eye care related information of PRIMARY RESPONDENT**

| **Section 3A: Primary respondent’s general information** | | |
| --- | --- | --- |
| Participant’s ID no: [__\|__\|__\|__\|__\|__] | Participant’s Name: | |
| Age: [__\|__] years - [__\|__] months | Gender: Male = 1 Female = 2 | [__\|__] |
| Patient’s Occupation: | Patient’s Religion: | |

| **Section 3B: Current Eye Problem** | | | | | | | |
| --- | --- | --- | --- | --- | --- | --- | --- |
| **Code** | **Question** | | **Response Code** | | | **Answer** | |
| Q3B_01 | Do you have any current eye problem? | | Yes = 01 No = 02 | | | [__\|__] | |
| (*If “No” go to next Section, if “Yes” proceed*) | | | | | | | |
| Q3B_02 | **What is/are the eye problem/s you have right now? (Can be multiple responses).** | | | | | 1^st^Problem - [__\|__]  2^nd^ Problem -[__\|__]  3^rd^ Problem - [__\|__] | |
|  | Lacrimation = 01  Discharge = 02  Itching/ Irritation = 03  Burning sensation = 04  Dry eye = 05  Eye ache = 06  Conjunctivitis = 07 | Photophobia = 08  Eye trauma = 09  Swelling of Eyelids = 10  Corneal Opacity = 11  Distance vision = 12  Near vision = 13  Blurred of vision =14 | | | | Low vision at night = 15  Blindness = 16  Cataract = 17  Squint = 18  Other = 19 (Please Specify)  [­­­­­­­­­­­­­­­­­­­­_________________] | |
| Q3B_03 | **How long have you been suffering from this/these problem/s?** | | | | 1^st^Problem - [__\|__] y [__\|__] m [__\|__] d  2^nd^ Problem -[__\|__] y [__\|__] m [__\|__] d    3^rd^ Problem - [__\|__] y [__\|__] m [__\|__] d | | |
| Q3B_04 | **What did you do for the treatment of your current eye problem/s?** | | | Nothing = 00  Used home remedy = 01  When to a health care provider = 02 | | | 1^st^ Problem -[__\|__]  2^nd^ Problem -[__\|__]  3^rd^ Problem -[__\|__] |
| (*If the answer is “0” or “1” go to next Section, if “2” proceed*) | | | | | | | |
| Q3B_05 | **From what kind of service provider did you seek eye treatment from?** | | | | | | 1^st^ Problem - [__\|__]  2^nd^ Problem -[__\|__]  3^rd^ Problem -[__\|__] |
|  | MBBS Doctor = 01  Non- MBBS Doctor = 02  Health Worker = 03 | NGO Hospital = 04  Private clinic = 05  Public Hospital = 06 | | | | | Pharmacy = 07  Eye Camp = 08  Traditional Healer = 09  Other = 10 (Please Specify)  [­­­­­­­­­­­­­­­­­­­­­­­­­­­­_________________] |
| Q3B_06 | **How long did you take to go the health provider after you got the eye problem/s?** | | | | 1^st^Problem - [__\|__] y [__\|__] m [__\|__] d  2^nd^ Problem -[__\|__] y [__\|__] m [__\|__] d    3^rd^ Problem - [__\|__] y [__\|__] m [__\|__] d | | |

| **Section 3C: Past Eye Problem (Within last 30 Days)** | | | | | | | |
| --- | --- | --- | --- | --- | --- | --- | --- |
| **Code** | **Question** | | **Response Code** | | | **Answer** | |
| Q3C_01 | **Did you suffer from any eye problem within last 30 days (which you do not have right now)?** | | Yes = 01 No = 02 | | | [__\|__] | |
| (*If “No” go to next Section, if “Yes” proceed*) | | | | | | | |
| Q3C_02 | **What was/were the eye problem/s that you suffered from in last 30 days? (Can be multiple responses).** | | | | | 1^st^Problem - [__\|__]  2^nd^ Problem -[__\|__]  3^rd^ Problem - [__\|__] | |
|  | Lacrimation = 01  Discharge = 02  Itching/ Irritation = 03  Burning sensation = 04  Dry eye = 05  Eye ache = 06  Conjunctivitis = 07 | Photophobia = 08  Eye trauma = 09  Swelling of Eyelids = 10  Corneal Opacity = 11  Distance vision = 12  Near vision = 13  Blurred of vision =14 | | | | Low vision at night = 15  Blindness = 16  Cataract = 17  Squint = 18  Other = 19 (Please Specify)  [­­­­­­­­­­­­­­­­­­­­_________________] | |
| Q3C_03 | **How long did you suffer from this/these problem/s?** | | | | 1^st^Problem - [__\|__] y [__\|__] m [__\|__] d  2^nd^ Problem -[__\|__] y [__\|__] m [__\|__] d    3^rd^ Problem - [__\|__] y [__\|__] m [__\|__] d | | |
| Q3C_04 | **What did you do for the treatment of this/these eye problem/s?** | | | Nothing = 00  Used home remedy = 01  When to a health care provider = 02 | | | 1^st^ Problem -[__\|__]  2^nd^ Problem -[__\|__]  3^rd^ Problem -[__\|__] |
| (*If the answer is “0” or “1” go to next Section, if “2” proceed*) | | | | | | | |
| Q3C_05 | **From what kind of service provider did you seek eye treatment from?** | | | | | | 1^st^ Problem - [__\|__]  2^nd^ Problem -[__\|__]  3^rd^ Problem -[__\|__] |
|  | MBBS Doctor = 01  Non- MBBS Doctor = 02  Health Worker = 03 | NGO Hospital = 04  Private clinic = 05  Public Hospital = 06 | | | | | Pharmacy = 07  Eye Camp = 08  Traditional Healer = 09  Other = 10 (Please Specify)  [­­­­­­­­­­­­­­­­­­­­­­­­­­­­_________________] |
| Q3C_06 | **How long did you take to go the health provider after you got the eye problem/s?** | | | | 1^st^Problem - [__\|__] y [__\|__] m [__\|__] d  2^nd^ Problem -[__\|__] y [__\|__] m [__\|__] d    3^rd^ Problem - [__\|__] y [__\|__] m [__\|__] d | | |

| **Section 3D: Family History of Eye Problem** | | | | | |
| --- | --- | --- | --- | --- | --- |
| Q3D_01 | **Does any of your family member has/had any kind of eye related problem/s?** (Grandparents/parents/siblings) | | Yes = 01 No = 02 | | [__\|__] |
| *(if “Yes” proceed, if “No”, go to the next section)* | | | | | |
| Q3D_02 | **What kind of eye problem/s did s/he/they have?**  *(Can be multiple response)* | | | | [__\|__]  [__\|__]  [__\|__] |
|  | Cataract = 01  Low vision (for near object) = 02  Low vision (for far object) = 03 | Glaucoma = 04  Night Blindness = 05  Nystigmus = 06 | | Strabismus = 07  Colour blindness= 08  Other = 08 (Please Specify)  [­­­­­­­­­­­­­­­­­­­­­­­­­­­­_________________] | |

| **Section 3E: External Risk factor** | | | | |
| --- | --- | --- | --- | --- |
| Q3E_01 | **Are you exposed to sun everyday due to your work/ daily routine?** | Yes = 01 No = 02 | | [__\|__] |
| *(if “Yes” proceed, if “No”, go to Q3E_03)* | | | | |
| Q3E_02 | **For how many hours you are exposed to sun every day?** | | | [__\|__] hour/s |
| Q3E_03 | **Have you ever smoked cigarettes or biris?** | Yes = 01 No = 02 | | [__\|__] |
| *(if “Yes” proceed, if “No”, go to the next section)* | | | | |
| Q3E_04 | **Do you currently smoke cigarette or biri?** | Yes = 01 No = 02 | | [__\|__] |
| *(if “Yes” proceed, if, “No” go to Q3E_07)* | | | | |
| Q3E_05 | **How long have you been smoking cigarette or biri?** | | [__\|__] y [__\|__] m [__\|__] d | |
| Q3E_06 | **On an average how many sticks do / did you smoke per day?** | | | [__\|__]sticks/ day |
| Q3E_07 | **How long has it been since you quit smoking?** | | [__\|__] y [__\|__] m [__\|__] d | |

| **Section 3F: Internal Risk Factors** | | | | | | |
| --- | --- | --- | --- | --- | --- | --- |
| Q3F_01 | **Have you ever had an eye injury for which you needed to seek care for?** | | Yes = 01 No = 02 | | | [__\|__] |
| *(if “Yes” proceed, if “No”, go to Q3F_05)* | | | | | | |
| Q3F_02 | **How many times you have experienced major eye injury in your lifetime?** | | | | | [__\|__] times |
| Q3F_03 | **How long has it been since your last eye injury?** | | | | [__\|__] y [__\|__] m [__\|__] d | |
| Q3F_04 | **From what kind of service provider did you seek eye treatment for the last eye injury?** | | | | | [__\|__] |
|  | MBBS Doctor = 01  Non- MBBS Doctor = 02  Health Worker = 03  NGO = 04 | Private clinic = 05  Public Hospital = 06  Pharmacy = 07  Eye Camp = 08 | | Traditional Healer = 09  Did not seek treatment = 10  Home remedy = 11  Other = 12 (Please Specify)  [­­­­­­­­­­­­­­­­­­­­­­­­­­­­_________________] | | |
| Q3F_05 | **Have you ever had an eye infection/ inflammation?** | | Yes = 01 No = 02 | | | [__\|__] |
| *(if “Yes” proceed, if “No”, go to Q3F_09)* | | | | | | |
| Q3F_06 | **How many times you have experienced major eye infection/ inflammation in your lifetime?** | | | | | [__\|__] times |
| Q3F_07 | **How long has it been since your last eye infection/ inflammation?** | | | | [__\|__] y [__\|__] m [__\|__] d | |
| Q3F_08 | **From what kind of service provider did you seek eye treatment for the last eye infection/ inflammation?** | | | | | [__\|__] |
|  | MBBS Doctor = 01  Non- MBBS Doctor = 02  Health Worker = 03  NGO = 04 | Private clinic = 05  Public Hospital = 06  Pharmacy = 07  Eye Camp = 08 | | Traditional Healer = 09  Did not seek treatment = 10  Home remedy = 11  Other = 12 (Please Specify)  [­­­­­­­­­­­­­­­­­­­­­­­­­­­­_________________] | | |

| Q3F_09 | **Have you ever had an eye surgery?** | | Yes = 01 No = 02 | | | | [__\|__] |
| --- | --- | --- | --- | --- | --- | --- | --- |
| *(if “Yes” proceed, if “No”, go to the next section)* | | | | | | | |
| Q3F_10 | **How many times did you go through eye surgery in your lifetime?** | | | | | | [__\|__] times |
| Q3F_11 | **How long has it been since your last eye surgery?** | | | | [__\|__] y [__\|__] m [__\|__] d | | |
| Q3F_12 | **What was the reason for your last surgery?** | | | | [__\|__] | | |
|  | Cataract = 01  Keratoconus = 02 | Glucoma = 03  Diabetes retinopathy = 04 | | | | Correction of refraction error = 05  Other = 06 (Please Specify)  [­­­­­­­­­­­­­­­­­­­­­­­­­­­­_________________] | |
| Q3F_13 | **From what kind of service provider did you seek eye treatment for the last eye surgery?** | | | | | | [__\|__] |
|  | Public Hospital = 01  Private Hospital = 02 | Eye Camp = 03  Voluntary/charitable hospital = 04 | | Other = 05 (Please Specify)  [­­­­­­­­­­­­­­­­­­­­­­­­­­­­_________________] | | | |

| **Section 3G: Medical History** | | | | |
| --- | --- | --- | --- | --- |
| Q3G_01 | **Do you have high blood pressure?** | Yes = 01 No = 02 | | [__\|__] |
| *(if “Yes” proceed, if “No”, go to “Q3G_05”)* | | | | |
| Q3G_02 | **For how long have you had high blood pressure?** | | | [__\|__]years- [__\|__]- months |
| Q3G_03 | **Are you taking any medication for it?** | Yes = 01 No = 02 | | [__\|__] |
| *(if “Yes” proceed, if “No”, go to “Q3G_05”)* | | | | |
| Q3G_04 | **How long have you been taking medication for your high blood pressure?** | | [__\|__] y [__\|__] m [__\|__] d | |

| Q3G_05 | **Do you have diabetes?** | Yes = 01 No = 02 | | [__\|__] |
| --- | --- | --- | --- | --- |
| *(if “Yes” proceed, if “No”, go to next “Q3G_09”)* | | | | |
| Q3G_06 | **For how long have you had diabetes?** | | | [__\|__] years- [__\|__]- months |
| Q3G_07 | **Are you taking any medication for it?** | Yes = 01 No = 02 | | [__\|__] |
| Q3G_08 | **How long have you been taking medication for your diabetes?** | | [__\|__] y [__\|__] m [__\|__] d | |

| Q3G_09 | **Have you been taking any medication/s for last three months or more** | Yes = 01 No = 02 | | [__\|__] |
| --- | --- | --- | --- | --- |
| *(if “Yes” proceed, if “No” finish the interview)* | | | | |
| Q3G_10 | **Can you please name or show the medication/s?**  *(If participant can’t remember the name, tell him/her to show the medication/s)* | | 1.[­­­­­­­­­­­­­­­­­­­­­­­­­­­­_________________]  2.[­­­­­­­­­­­­­­­­­­­­­­­­­­­­_________________]  3.[­­­­­­­­­­­­­­­­­­­­­­­­­­­­_________________]  4.[­­­­­­­­­­­­­­­­­­­­­­­­­­­­_________________] | |
| Q3G_11 | **How long have you been under this/these medication/s?** | Medication No. 1 | [__\|__] years- [__\|__]- months | |
|  |  | Medication No. 2 | [__\|__] years- [__\|__]- months | |
|  |  | Medication No. 3 | [__\|__] years- [__\|__]- months | |
|  |  | Medication No. 4 | [__\|__] years- [__\|__]- months | |

***If the primary respondent is ≥30 years, give him/her the Referral Slip***

***If the primary respondent is 18-29 year and reported any eye problem, give him/her the Referral Slip***

***If the respondent is eligible of getting the referral slip please counsel him/her to follow the next step of the research purpose***

***Please fill up the second part of the questionnaire by interviewing the secondary respondent***

**Section 04: Eye care related information of SECONDARY RESPONDENT**

| **Section 4A: Secondary respondent’s general information** | | |
| --- | --- | --- |
| Participant’s ID no: [__\|__\|__\|__\|__\|__] | Participant’s Name: | |
| Age: [__\|__] years - [__\|__] months | Gender: Male = 1 Female = 2 | [__\|__] |
| Patient’s Occupation: | Patient’s Religion: | |

| **Section 4B: Current Eye Problem** | | | | | | | |
| --- | --- | --- | --- | --- | --- | --- | --- |
| **Code** | **Question** | | **Response Code** | | | **Answer** | |
| Q4B_01 | Do you have any current eye problem? | | Yes = 01 No = 02 | | | [__\|__] | |
| (*If “No” go to next Section, if “Yes” proceed*) | | | | | | | |
| Q4B_02 | **What is/are the eye problem/s you have right now? (Can be multiple responses).** | | | | | 1^st^Problem - [__\|__]  2^nd^ Problem -[__\|__]  3^rd^ Problem - [__\|__] | |
|  | Lacrimation = 01  Discharge = 02  Itching/ Irritation = 03  Burning sensation = 04  Dry eye = 05  Eye ache = 06  Conjunctivitis = 07 | Photophobia = 08  Eye trauma = 09  Swelling of Eyelids = 10  Corneal Opacity = 11  Distance vision = 12  Near vision = 13  Blurred of vision =14 | | | | Low vision at night = 15  Blindness = 16  Cataract = 17  Squint = 18  Other = 19 (Please Specify)  [­­­­­­­­­­­­­­­­­­­­_________________] | |
| Q4B_03 | **How long have you been suffering from this/these problem/s?** | | | | 1^st^Problem - [__\|__] y [__\|__] m [__\|__] d  2^nd^ Problem -[__\|__] y [__\|__] m [__\|__] d    3^rd^ Problem - [__\|__] y [__\|__] m [__\|__] d | | |
| Q4B_04 | **What did you do for the treatment of your current eye problem/s?** | | | Nothing = 00  Used home remedy = 01  When to a health care provider = 02 | | | 1^st^ Problem -[__\|__]  2^nd^ Problem -[__\|__]  3^rd^ Problem -[__\|__] |
| (*If the answer is “0” or “1” go to next Section, if “2” proceed*) | | | | | | | |
| Q4B_05 | **From what kind of service provider did you seek eye treatment from?** | | | | | | 1^st^ Problem - [__\|__]  2^nd^ Problem -[__\|__]  3^rd^ Problem -[__\|__] |
|  | MBBS Doctor = 01  Non- MBBS Doctor = 02  Health Worker = 03 | NGO Hospital = 04  Private clinic = 05  Public Hospital = 06 | | | | | Pharmacy = 07  Eye Camp = 08  Traditional Healer = 09  Other = 10 (Please Specify)  [­­­­­­­­­­­­­­­­­­­­­­­­­­­­_________________] |
| Q4B_06 | **How long did you take to go the health provider after you got the eye problem/s?** | | | | 1^st^Problem - [__\|__] y [__\|__] m [__\|__] d  2^nd^ Problem -[__\|__] y [__\|__] m [__\|__] d    3^rd^ Problem - [__\|__] y [__\|__] m [__\|__] d | | |

| **Section 3C: Past Eye Problem (Within last 30 Days)** | | | | | | | |
| --- | --- | --- | --- | --- | --- | --- | --- |
| **Code** | **Question** | | **Response Code** | | | **Answer** | |
| Q4C_01 | **Did you suffer from any eye problem within last 30 days (which you do not have right now)?** | | Yes = 01 No = 02 | | | [__\|__] | |
| (*If “No” go to next Section, if “Yes” proceed*) | | | | | | | |
| Q4C_02 | **What was/were the eye problem/s that you suffered from in last 30 days? (Can be multiple responses).** | | | | | 1^st^Problem - [__\|__]  2^nd^ Problem -[__\|__]  3^rd^ Problem - [__\|__] | |
|  | Lacrimation = 01  Discharge = 02  Itching/ Irritation = 03  Burning sensation = 04  Dry eye = 05  Eye ache = 06  Conjunctivitis = 07 | Photophobia = 08  Eye trauma = 09  Swelling of Eyelids = 10  Corneal Opacity = 11  Distance vision = 12  Near vision = 13  Blurred of vision =14 | | | | Low vision at night = 15  Blindness = 16  Cataract = 17  Squint = 18  Other = 19 (Please Specify)  [­­­­­­­­­­­­­­­­­­­­_________________] | |
| Q4C_03 | **How long did you suffer from this/these problem/s?** | | | | 1^st^Problem - [__\|__] y [__\|__] m [__\|__] d  2^nd^ Problem -[__\|__] y [__\|__] m [__\|__] d    3^rd^ Problem - [__\|__] y [__\|__] m [__\|__] d | | |
| Q4C_04 | **What did you do for the treatment of this/these eye problem/s?** | | | Nothing = 00  Used home remedy = 01  When to a health care provider = 02 | | | 1^st^ Problem -[__\|__]  2^nd^ Problem -[__\|__]  3^rd^ Problem -[__\|__] |
| (*If the answer is “0” or “1” go to next Section, if “2” proceed*) | | | | | | | |
| Q4C_05 | **From what kind of service provider did you seek eye treatment from?** | | | | | | 1^st^ Problem - [__\|__]  2^nd^ Problem -[__\|__]  3^rd^ Problem -[__\|__] |
|  | MBBS Doctor = 01  Non- MBBS Doctor = 02  Health Worker = 03 | NGO Hospital = 04  Private clinic = 05  Public Hospital = 06 | | | | | Pharmacy = 07  Eye Camp = 08  Traditional Healer = 09  Other = 10 (Please Specify)  [­­­­­­­­­­­­­­­­­­­­­­­­­­­­_________________] |
| Q4C_06 | **How long did you take to go the health provider after you got the eye problem/s?** | | | | 1^st^Problem - [__\|__] y [__\|__] m [__\|__] d  2^nd^ Problem -[__\|__] y [__\|__] m [__\|__] d    3^rd^ Problem - [__\|__] y [__\|__] m [__\|__] d | | |

| **Section 3D: Family History of Eye Problem** | | | | | |
| --- | --- | --- | --- | --- | --- |
| Q4D_01 | **Does any of your family member has/had any kind of eye related problem/s?** (Grandparents/parents/siblings) | | Yes = 01 No = 02 | | [__\|__] |
| *(if “Yes” proceed, if “No”, go to the next section)* | | | | | |
| Q4D_02 | **What kind of eye problem/s did s/he/they have?**  *(Can be multiple response)* | | | | [__\|__]  [__\|__]  [__\|__] |
|  | Cataract = 01  Low vision (for near object) = 02  Low vision (for far object) = 03 | Glaucoma = 04  Night Blindness = 05  Nystigmus = 06 | | Strabismus = 07  Colour blindness= 08  Other = 08 (Please Specify)  [­­­­­­­­­­­­­­­­­­­­­­­­­­­­_________________] | |

| **Section 3E: External Risk factor** | | | | |
| --- | --- | --- | --- | --- |
| Q4E_01 | **Are you exposed to sun everyday due to your work/ daily routine?** | Yes = 01 No = 02 | | [__\|__] |
| *(if “Yes” proceed, if “No”, go to Q4E_03)* | | | | |
| Q4E_02 | **For how many hours you are exposed to sun every day?** | | | [__\|__] hour/s |
| Q4E_03 | **Have you ever smoked cigarettes or biris?** | Yes = 01 No = 02 | | [__\|__] |
| *(if “Yes” proceed, if “No”, go to the next section)* | | | | |
| Q4E_04 | **Do you currently smoke cigarette or biri?** | Yes = 01 No = 02 | | [__\|__] |
| *(if “Yes” proceed, if, “No” go to Q4E_07)* | | | | |
| Q4E_05 | **How long have you been smoking cigarette or biri?** | | [__\|__] y [__\|__] m [__\|__] d | |
| Q4E_06 | **On an average how many sticks do / did you smoke per day?** | | | [__\|__]sticks/ day |
| Q4E_07 | **How long has it been since you quit smoking?** | | [__\|__] y [__\|__] m [__\|__] d | |

| **Section 3F: Internal Risk Factors** | | | | | | |
| --- | --- | --- | --- | --- | --- | --- |
| Q4F_01 | **Have you ever had an eye injury for which you needed to seek care for?** | | Yes = 01 No = 02 | | | [__\|__] |
| *(if “Yes” proceed, if “No”, go to Q4F_05)* | | | | | | |
| Q4F_02 | **How many times you have experienced major eye injury in your lifetime?** | | | | | [__\|__] times |
| Q4F_03 | **How long has it been since your last eye injury?** | | | | [__\|__] y [__\|__] m [__\|__] d | |
| Q4F_04 | **From what kind of service provider did you seek eye treatment for the last eye injury?** | | | | | [__\|__] |
|  | MBBS Doctor = 01  Non- MBBS Doctor = 02  Health Worker = 03  NGO = 04 | Private clinic = 05  Public Hospital = 06  Pharmacy = 07  Eye Camp = 08 | | Traditional Healer = 09  Did not seek treatment = 10  Home remedy = 11  Other = 12 (Please Specify)  [­­­­­­­­­­­­­­­­­­­­­­­­­­­­_________________] | | |
| Q4F_05 | **Have you ever had an eye infection/ inflammation?** | | Yes = 01 No = 02 | | | [__\|__] |
| *(if “Yes” proceed, if “No”, go to Q4F_09)* | | | | | | |
| Q4F_06 | **How many times you have experienced major eye infection/ inflammation in your lifetime?** | | | | | [__\|__] times |
| Q4F_07 | **How long has it been since your last eye infection/ inflammation?** | | | | [__\|__] y [__\|__] m [__\|__] d | |
| Q4F_08 | **From what kind of service provider did you seek eye treatment for the last eye infection/ inflammation?** | | | | | [__\|__] |
|  | MBBS Doctor = 01  Non- MBBS Doctor = 02  Health Worker = 03  NGO = 04 | Private clinic = 05  Public Hospital = 06  Pharmacy = 07  Eye Camp = 08 | | Traditional Healer = 09  Did not seek treatment = 10  Home remedy = 11  Other = 12 (Please Specify)  [­­­­­­­­­­­­­­­­­­­­­­­­­­­­_________________] | | |

| Q4F_09 | **Have you ever had an eye surgery?** | | Yes = 01 No = 02 | | | | [__\|__] |
| --- | --- | --- | --- | --- | --- | --- | --- |
| *(if “Yes” proceed, if “No”, go to the next section)* | | | | | | | |
| Q4F_10 | **How many times did you go through eye surgery in your lifetime?** | | | | | | [__\|__] times |
| Q4F_11 | **How long has it been since your last eye surgery?** | | | | [__\|__] y [__\|__] m [__\|__] d | | |
| Q4F_12 | **What was the reason for your last surgery?** | | | | [__\|__] | | |
|  | Cataract = 01  Keratoconus = 02 | Glucoma = 03  Diabetes retinopathy = 04 | | | | Correction of refraction error = 05  Other = 06 (Please Specify)  [­­­­­­­­­­­­­­­­­­­­­­­­­­­­_________________] | |
| Q4F_13 | **From what kind of service provider did you seek eye treatment for the last eye surgery?** | | | | | | [__\|__] |
|  | Public Hospital = 01  Private Hospital = 02 | Eye Camp = 03  Voluntary/charitable hospital = 04 | | Other = 05 (Please Specify)  [­­­­­­­­­­­­­­­­­­­­­­­­­­­­_________________] | | | |

| **Section 3G: Medical History** | | | | |
| --- | --- | --- | --- | --- |
| Q4G_01 | **Do you have high blood pressure?** | Yes = 01 No = 02 | | [__\|__] |
| *(if “Yes” proceed, if “No”, go to “Q4G_05”)* | | | | |
| Q4G_02 | **For how long have you had high blood pressure?** | | | [__\|__]years- [__\|__]- months |
| Q4G_03 | **Are you taking any medication for it?** | Yes = 01 No = 02 | | [__\|__] |
| *(if “Yes” proceed, if “No”, go to “Q4G_05”)* | | | | |
| Q4G_04 | **How long have you been taking medication for your high blood pressure?** | | [__\|__] y [__\|__] m [__\|__] d | |

| Q4G_05 | **Do you have diabetes?** | Yes = 01 No = 02 | | [__\|__] |
| --- | --- | --- | --- | --- |
| *(if “Yes” proceed, if “No”, go to next “Q4G_09”)* | | | | |
| Q4G_06 | **For how long have you had diabetes?** | | | [__\|__] years- [__\|__]- months |
| Q4G_07 | **Are you taking any medication for it?** | Yes = 01 No = 02 | | [__\|__] |
| Q4G_08 | **How long have you been taking medication for your diabetes?** | | [__\|__] y [__\|__] m [__\|__] d | |

| Q4G_09 | **Have you been taking any medication/s for last three months or more** | Yes = 01 No = 02 | | [__\|__] |
| --- | --- | --- | --- | --- |
| *(if “Yes” proceed, if “No” finish the interview)* | | | | |
| Q4G_10 | **Can you please name or show the medication/s?**  *(If participant can’t remember the name, tell him/her to show the medication/s)* | | 1.[­­­­­­­­­­­­­­­­­­­­­­­­­­­­_________________]  2.[­­­­­­­­­­­­­­­­­­­­­­­­­­­­_________________]  3.[­­­­­­­­­­­­­­­­­­­­­­­­­­­­_________________]  4.[­­­­­­­­­­­­­­­­­­­­­­­­­­­­_________________] | |
| Q4G_11 | **How long have you been under this/these medication/s?** | Medication No. 1 | [__\|__] years- [__\|__]- months | |
|  |  | Medication No. 2 | [__\|__] years- [__\|__]- months | |
|  |  | Medication No. 3 | [__\|__] years- [__\|__]- months | |
|  |  | Medication No. 4 | [__\|__] years- [__\|__]- months | |

***If the primary respondent is ≥30 years, give him/her the Referral Slip***

***If the primary respondent is 18-29 year and reported any eye problem, give him/her the Referral Slip***

***If the respondent is eligible of getting the referral slip please counsel him/her to follow the next step of the research purpose***

***Thank you***
