# Supplementary material for: Eye diseases: the neglected health condition among urban slum population of Dhaka, Bangladesh
Source: BMC Ophthalmol. 2019 Jan 31;19:38. doi: 10.1186/s12886-019-1043-z (PMC6357461; doi:10.1186/s12886-019-1043-z)
Supplement: Supplementary file 2 — Clinical examination form This form was used to collect data on participants’ eye examination result and clinical diagnosis of eye disease. (DOCX 1713 kb) [file 12886_2019_1043_MOESM2_ESM.docx]

CLINICAL EXAMINATION FORM

participant informed Consent FORM

**eye examination**

**Accessibility of Eye Care Service in an Urban Setting in Bangladesh**

Dear Participant,

Greetings, I am _________________________ and this is *(If there are two interviewers)*. I am/we are working in a research project under BRAC James P Grant School of Public Health, BRAC University.

We are conducting a study on prevalence of eye problems in the community and heath seeking behavior for eye problems. In this study we hope to learn the prevalence of different eye problems existing in different age groups and where people seek eye care for these problems. For this purpose, we provided a free eye check-up ticket to all participants of household survey having age 18 years or more. You are one of the participants who took part in household survey. We would like to thank you for coming to this hospital for eye check-up in response to our request. Our team members will collect your personal information. Doctors (Ophthalmologists) of this hospital will examine your eyes and will advice for laboratory investigation if necessary.

All the cost of eye examination and necessary laboratory investigations will be offered by BRAC James P Grant School of Public Health, BRAC University. This will include only diagnosis of your eye problem. If you are diagnosed having any eye disease, doctor will prescribe medication to you and will counsel you. However, BRAC James P Grant School of Public Health will not offer any monetary support for treatment of your eye disease.

You will be provided with 200 Taka as travel expense for attending hospital. You will be able to collect this amount after showing necessary document on eye examination and laboratory investigation to our team members at hospital.

Your personal detail and result of your eye examination will be kept confidential and will not be used for any other purpose other than research. You can ask for any clarification regarding this research and can withdraw yourself at any stage of eye examination. It will not affect you in future for receiving any kind of health service from this hospital.

Do you have any further inquiry about this study? Yes No

(*If “Yes” please answer to any other inquiry of the participant)*

Do you agree to participate in this study? Yes No

| Signature of the participants: | Name of the participants: | Date: |
| --- | --- | --- |

I have provided all the necessary information to the participant and he/she agreed to take part in eye examination process voluntarily.

| Signature of the doctor: | Name of the doctor: | Date: |
| --- | --- | --- |

| **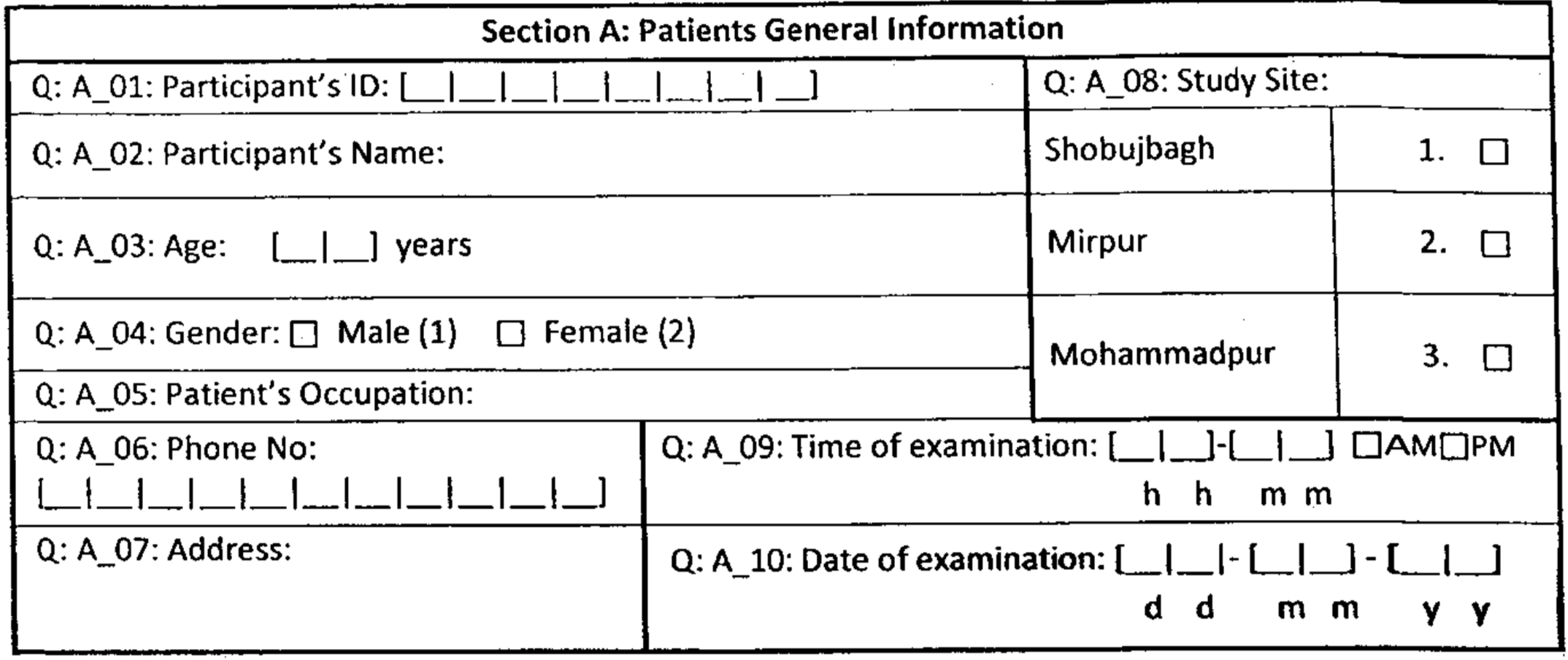** |
| --- |
| **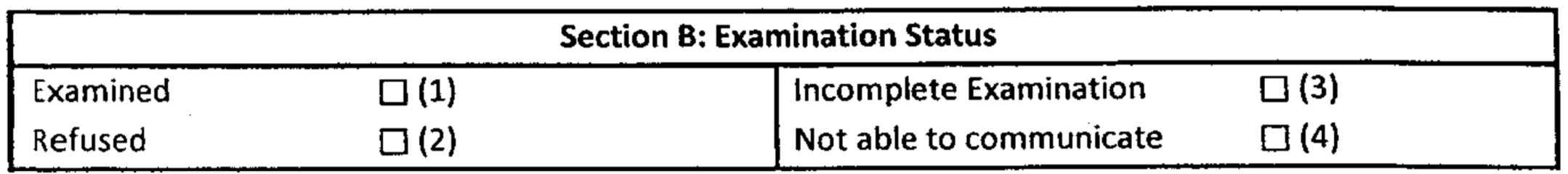** |
| **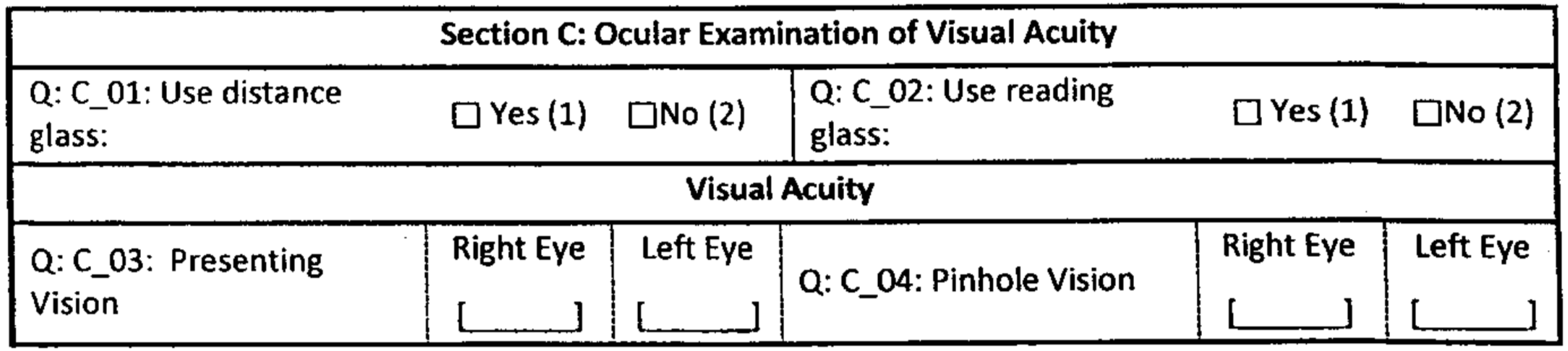** |
| **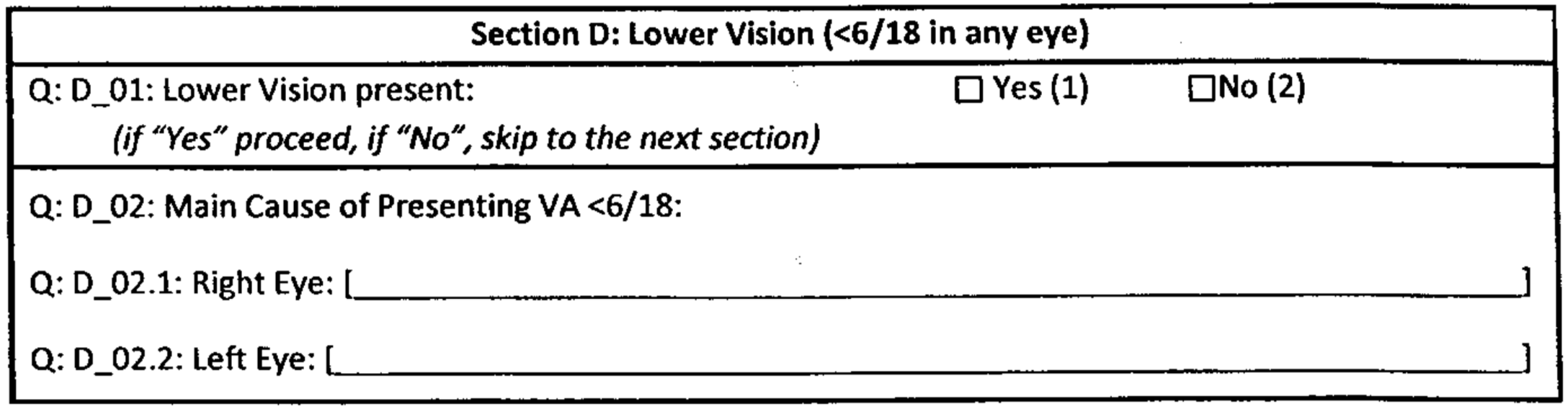** |
| **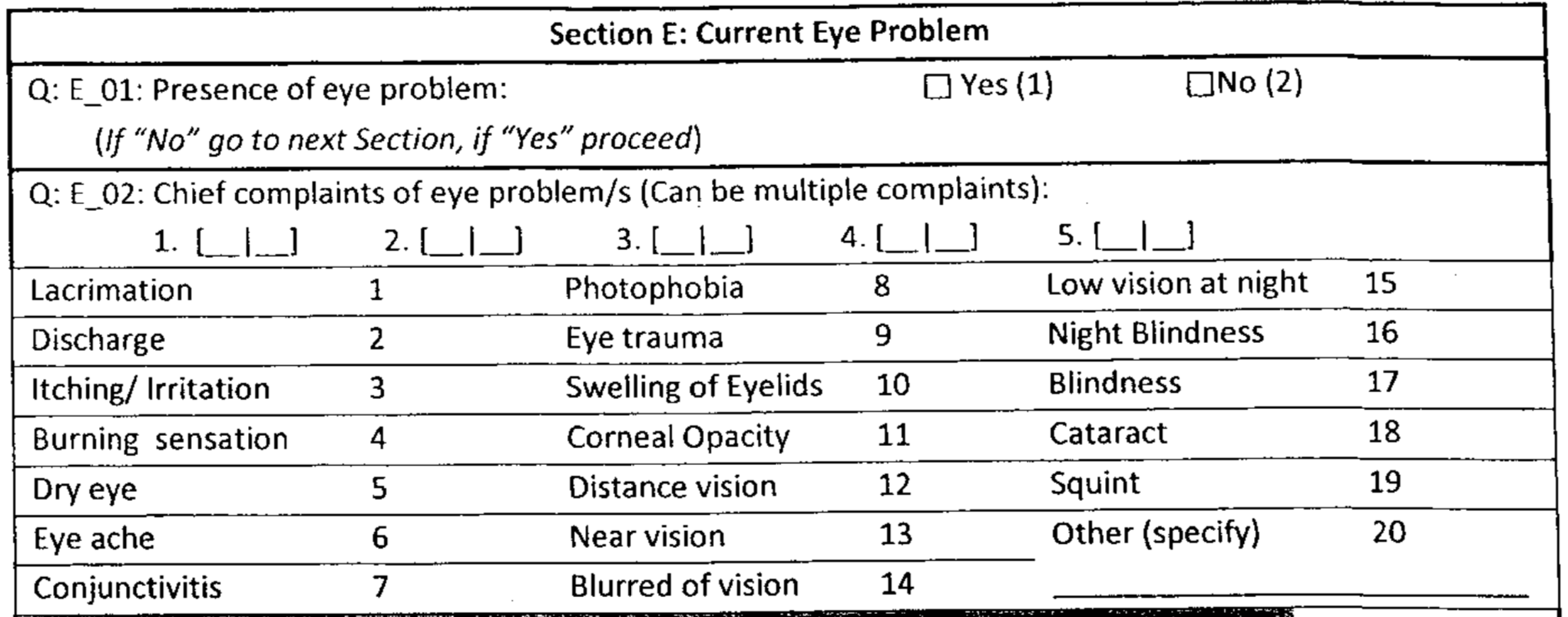** |
| 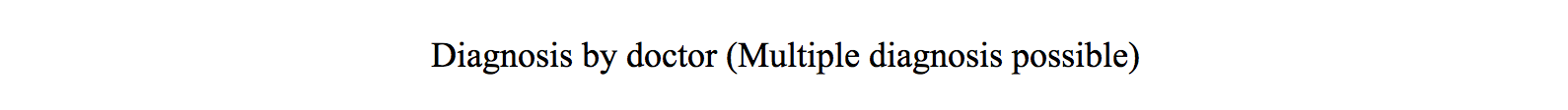 |
| **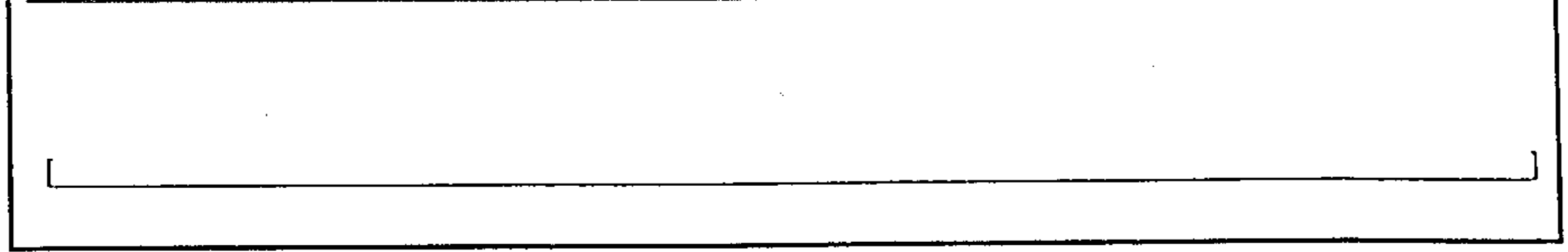** |
| **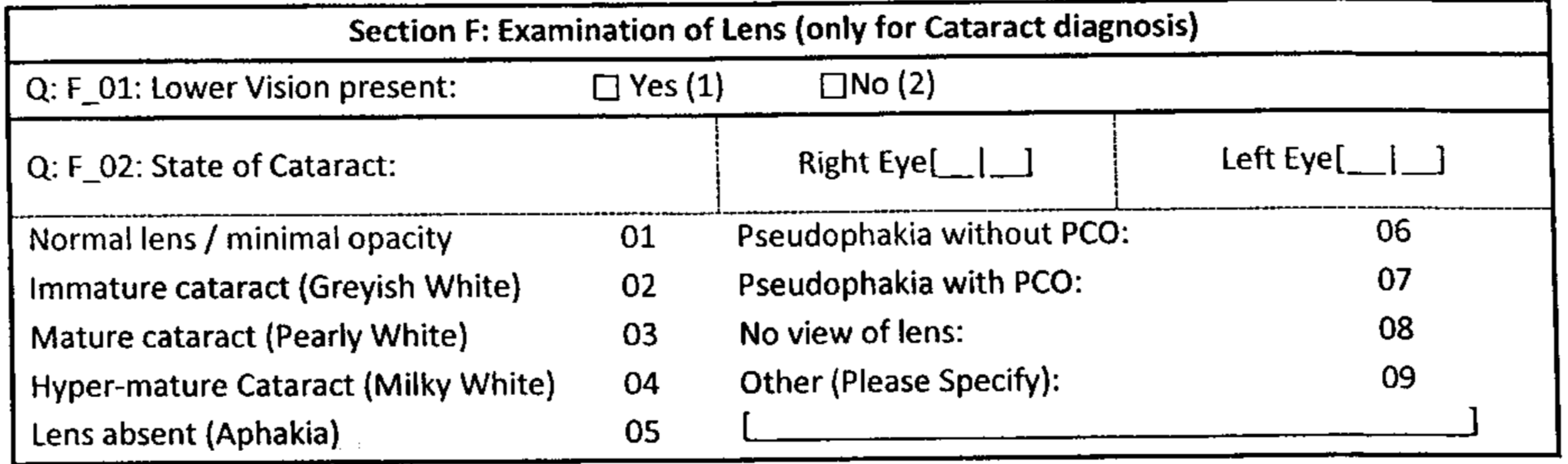** |
